# SARS-CoV-2 Infection in Cancer Patients: A Population-Based Study

**DOI:** 10.3389/fonc.2021.730131

**Published:** 2021-10-11

**Authors:** Manuel Zorzi, Stefano Guzzinati, Francesco Avossa, Ugo Fedeli, Arianna Calcinotto, Massimo Rugge

**Affiliations:** ^1^ Regional Epidemiological Service Unit, Azienda Zero, Padova, Italy; ^2^ Veneto Tumor Registry, Azienda Zero, Padova, Italy; ^3^ Institute of Oncology Research (iOR), Oncology Institute of Southern Switzerland, Bellinzona, Switzerland; ^4^ Department of Medicine - DIMED, Surgical Pathology and Cytopathology Unit, Università degli Studi di Padova, Padova, Italy

**Keywords:** SARS- CoV-2, neoplasms, cohort study, mortality rate, comorbidities

## Abstract

**Aim:**

In a consecutive series of cancer patients tested for SARS-CoV-2 infection, this retrospective population-based study investigates the risks of viral infection and death.

**Methods:**

Malignancies were distinguished as incident or prevalent (active or inactive). Cancer management and vital status were retrieved from institutional regional databases. Comorbidities were recorded, based on Adjusted Clinical Groups (ACG). Six Resource Utilization Bands (RUBs) were also considered. Independent risk factors for SARS-CoV-2 infection and death were identified using multivariable logistic regression, considering sex, age, comorbidities and RUBs, cancer status (active *versus* prevalent), primary cancer site, and treatments (chemotherapy and/or radiotherapy).

**Results:**

Among 34,929 cancer patients, 1,090 (3.1%) tested positive for SARS-CoV-2 infection (CoV2+*ve*). The risk of infection was associated with age (OR per 1-year increase=1.012; 95%CI=1.007-1.017), prevalent-inactive disease, hematologic malignancies (OR=1.33; 95%CI=1.03-1.72) and RUB (OR per 1-level increase=1.14; 95%CI=1.05-1.24). Among CoV2+*ve* cancer patients, the risk of death was doubled for males, and increased with age (OR per 1-year increase=1.07; 95%CI=1.06-1.09) and comorbidities (renal [OR=3.18; 95%CI=1.58-6.49], hematological [OR=3.08; 95%CI=1.49-6.50], respiratory [OR=2.87; 95%CI=1.61-5.14], endocrine [OR=2.09; 95%CI=1.25-3.51]). Lung and blood malignancies raised the mortality risk (OR=3.55; 95%CI=1.56-8.33, and OR=1.81; 95%CI=1.01-3.25 respectively). Incident or prevalent-active disease and recent chemotherapy and radiotherapy (OR=4.34; 95%CI=1.85-10.50) increased the risk of death.

**Conclusion:**

In a large cohort of cancer patients, the risk of SARS-CoV-2 infection was higher for those with inactive disease than in incident or prevalent-active cases. Among CoV2+ve cancer patients, active malignancies and recent multimodal therapy both significantly raised the risk of death, which increased particularly for lung cancer.

## Introduction

The epidemiology and clinical outcome of SARS-CoV-2 infection are both modulated (primarily) by several biological and clinical variables including viral biology, ethnicity, sex susceptibility, and patients’ comorbidities (1–3).

Cancer patients are considered prone to (mainly opportunistic) infectious diseases. This condition may be further promoted by several determinants, including immunocompetent status (primary and/or after anti-cancer therapies), time elapsing between the diagnosis of cancer and infection, and cancer biology, site and stage. Given this heterogeneous clinico-biological picture, the information available on the risk of SARS-CoV-2 infection and its clinical outcomes in cancer patients is still confusing (4–7).

While a number of valuable studies compared the risk of SARS-CoV-2 infection and its clinical outcomes in cancer *versus* non-cancer patients, few population studies focused specifically on cancer patients with *versus* without the viral infection to address the factors influencing the risk of contracting the virus and the clinical course of the viral disease (8–12).

Primary endpoints of this study were to assess the risks of viral infection and death in a cohort of consecutive cancer patients tested for SARS-CoV-2, considering demographics (age and sex), cancer-related variables (site of the primary malignancy, prevalent *versus* incident cancers, anticancer therapies), and comorbidities (as recorded according to Adjusted Clinical Groups [ACG]).

## Material and Methods

### Sources of Study Data

This retrospective population-based study considered a population of cancer patients resident in the Italian north-eastern Veneto Region and consecutively tested for SARS-CoV-2. Cancer patients were defined as individuals who received a diagnosis of malignancy within 10 years before testing for SARS-CoV-2.

**Figure 1:**
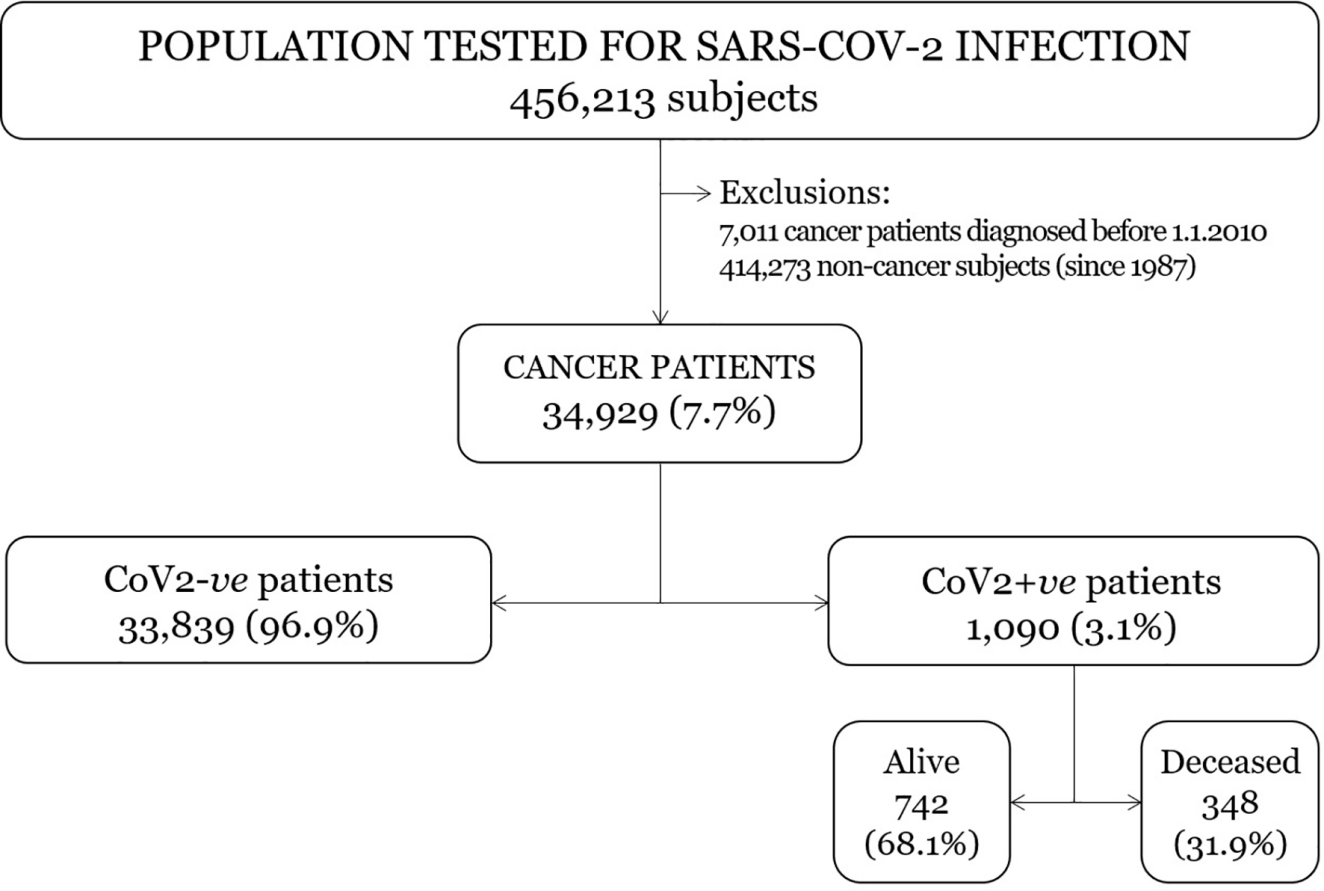
Flow chart of the study.

Among the regional residents tested for SARS-CoV-2 infection between February 22 and July 31, 2020 (456,213 in all; **Figure 1**), cancer patients were identified by linking the institutional SARS-CoV-2 test records with the Regional Cancer Registry database (**Figure 1**). In detail, patients diagnosed with cancer before December 31^st^, 2017 were identified from the Cancer Registry’s database. Due to the 3-year latency time in the (formal) cancer registration, cancer patients diagnosed from 2018 to 2020 were identified by merging the infromation achieved from both the databases of hospital discharge records (as available from the Regional/institutional health care system) and from the pathology reports (as available through the Regional/institutional archives of the Pathology Departments). Non-invasive solid malignancies and non-melanoma skin cancers were excluded.

Information on surgery, chemo-, and radio-therapy performed within 12 months before and/or after testing for SARS-CoV-2 was obtained through a linkage with the Regional Hospital Admissions and Outpatient Service Admissions databases (available up to 31 December 2020).

Comorbidities were recorded by applying the Johns Hopkins Adjusted Clinical Group (ACG) case-mix system to administrative data (hospital discharge records, outpatient service records, pharmaceutical prescriptions, access to emergency departments, prescription charge exemptions) (13).

The codes used to attribute clinically relevant comorbidities (“Expanded Diagnosis Clusters”) are listed in **Table A.1** (**Supplemetary Data**). Recorded comorbidities were not mutually exclusive. ACGs represent clinically logical categories for persons expected to require similar levels of healthcare resources (i.e.: resource groups). However, individuals with similar overall utilization may be assigned to different ACGs because of their different epidemiological patterns of morbidity. The ACG system allows collapsing the full set of ACGs into fewer categories (Resource Utilization Bands - RUBs) according to concurrent relative resource use. Cancer patients were then divided into six Resource Utilization Bands (RUB) ranging from 0 (Non-users) to 5 (Very High) by combining the mutually-exclusive ACG cells that measure overall morbidity burden (**Table A.2; Supplementary Data**) (13, 14).

Vital status was retrieved through record linkage with the Regional Health Service’s population lists as at 31 December 2020. Since information on cancer stage at diagnosis was only available for a subset of patients, this variable was not considered in the present analysis.

### Study Population

In the considered study population (**Figure 1**), viral status was always assessed using real-time PCR and next-generation sequencing, distinguishing between infected (CoV2+*ve*) and uninfected (CoV2-*ve*) cancer patients. The first test result was considered for individuals who had tested negative multiple times. Patients with alternately negative and positive test results were registered as CoV2+*ve* as at the time of their first positive test result.

Based on the time elapsing between SARS-CoV-2 testing and the patient’s latest cancer assessment and/or latest oncological therapy (surgery, chemotherapy or radiotherapy), “cancer status” was conventionally distinguished as follows: i) incident cancers (*i.e*., cancer patients diagnosed ≤12 months before testing for SARS-CoV-2); ii) prevalent-active cancers (*i.e*., cancer patients diagnosed >12 months before testing for SARS-CoV-2 who had received anticancer treatments within 12 months before and/or after testing for SARS-CoV-2); iii) prevalent-inactive cancers (*i.e*., cancer patients diagnosed >12 months before testing for SARS-CoV-2, who had received no anticancer treatments within 12 months before and/or after testing for SARS-CoV-2).

The follow-up time was calculated as the time elapsing between the date of viral testing and the end of the study.

### Statistical Analysis

This study considered a consecutive series of cancer patients tested for SARS-CoV-2 status to shed light on the variables associated with SARS-CoV-2 positivity. The cohort of CoV2+*ve* individuals was then examined to identify the determinants of any deaths.

The association between SARS-CoV-2 status and patients’ demographics and clinical profiles was examined with the chi-square test for proportions and the Mann-Whitney test for median age.

The associations between the study predictors and outcomes (SARS-CoV-2 positivity and death) were initially tested by computing prevalence Odds Ratios (with 95% Confidence Intervals) for sex, age, cancer status, primary cancer site, treatments performed within 12 months before and/or after SARS-CoV-2 testing, types of comorbidity, and RUB (15).

A multivariable logistic regression model (LRM) was then run to identify the factors associated with CoV2*+ve* status and death. All explanatory variables were included in the model.

The SAS EG v.6.1 (SAS Institute Inc., Cary, NC, USA) statistical package was used for all analyses. All statistical tests were two-tailed. A p-value <0.05 was considered statistically significant.

## Results

### The Study Cohort

Overall, during the study period 456,213 residents of the region were tested for SARS-CoV-2, and 19,359 of them tested positive (**Figure 1**). Among the population tested, the prevalence of patients with cancer detected during the 10 years before SARS-CoV-2 testing was 34,929/456,213. Among these cancer patients, some 1,090/34,929 were found CoV2+*ve* (3.1%) and 33,839 were CoV2-*ve* (96.9%).

**Table 1** shows demographics and clinical profiles of the study cohort, also distinguishing between CoV2*+ve* and CoV2*-ve* cancer patients. Males accounted for 50.3% overall, with similar proportions in the CoV2+*ve* (50.2%) and CoV2-*ve* (50.4%) subgroups. Median age was 70 years, and was significantly higher for the CoV2+*ve* (75 years) than for the CoV2-*ve* cancer patients (70 years; p<0.0001).

**Table 1:** Demographics and clinical profile of the study cohort.

Variables	Cohort of cancer patients		SARS-CoV-2 positive		SARS-CoV-2 negative		P value ^1^
	Number	%	Number	%	Number	%	
Total	34,929	100	1,090	100	33,839	100	–
Sex							
Male Female	17,584 17,345	50.3 49.7	543 547	50.2 49.8	17,041 16,798	50.4 49.6	0.72
Age (years)							
(median, IQR)	70	58-79	75	61-83	70	58-79	<0.0001
Type of comorbidity ^2^							
Cardiovascular Endocrine Neurological Respiratory Gastroenterological Renal Hematological Rheumatological Psychiatric Others ^3^	10,813 3,736 2,842 2,256 1,927 1,594 1,121 1,039 1,019 2,452	31.0 10.7 8.1 6.5 5.5 4.6 3.2 3.0 2.9 7.0	425 139 128 102 56 54 50 34 37 99	39.0 12.8 11.7 9.4 5.1 5.0 4.6 3.1 3.4 9.1	10,388 3,597 2,714 2,154 1,871 1,540 1,071 1,005 982 2,353	30.7 10.6 8.0 6.4 5.5 4.6 3.2 3.0 2.9 7.0	<0.0001 0.026 <0.0001 <0.001 0.577 0530 0.009 0.775 0.342 0.007
Resource Utilization Band (RUB)							
Non-users+Healthy users Low Moderate High Very high	3,711 2,955 18,989 6,146 3,128	10.6 8.5 54.4 17.6 9.0	61 64 590 220 155	5.6 5.9 54.1 20.2 14.2	3,650 2,891 18,399 5,926 2,973	10.8 8.5 54.4 17.5 8.8	<0.0001
Cancer status							
Incident	14,861	42.6	279	25.6	14,582	43.1	<0.0001
Prevalent-active	5,423	15.5	112	10.3	5,311	15.7	
Prevalent-inactive	14,645	41.9	699	64.1	13,946	41.2	
Cancer site							
Breast Urinary system Colon-rectum Prostate Blood Lung Others	6,441 4,876 3,658 3,452 3,101 1,934 11,467	18.4 14.0 10.5 9.9 8.9 5.5 32.8	183 127 137 134 124 45 340	16.8 11.7 12.6 12.3 11.4 4.1 31.2	6,258 4,749 3,521 3,318 2,977 1,889 11,127	18.5 14.0 10.4 9.8 8.8 5.6 32.9	<0.0001
Treatments ^4^							
None Chemotherapy only Radiotherapy only Chemo and radiotherapy	24,664 5,593 2,471 2,201	70.6 16.0 7.1 6.3	903 117 34 36	82.9 10.7 3.1 3.3	23,761 5,476 2,437 2,165	70.2 16.2 7.2 6.4	<0.0001
Vital status ^5^							
Alive Deceased	27,809 7,120	79.6 20.4	742 348	68.1 31.9	27,067 6,772	80.0 20.0	<0.0001
Time elapsing since viral testing							
Days	211 (170-248)		266 (88-283)		211 (170-246)		<0.0001

^1^ Chi-squared test for differences in distribution between SARS-CoV-2 positive and SARS-CoV-2 negative cases.

^2^ The categories are not mutually exclusives.

^3^ Patients with at least one comorbidity involving: toxic and adverse events, nutritional issues, infections, ocular, genetic, skin disorders, allergies.

^4^ Treatments performed ≤12 months before and/or after the date of testing for SARS-CoV-2 infection.

^5^ as at 31 December 2020.

Irrespective of SARS-CoV-2 status, the cancer patients’ most frequent comorbidities were: cardiovascular (31%); endocrine (10.7%) neurologic (8.1%); respiratory (6.5%); and gastrointestinal (5.5%). Univariate statistical analysis disclosed significantly higher proportions (p<o.0001) of comorbidities among CoV2+*ve* cancer patients, particularly for cardiovascular (39%), endocrine (12.8%) neurologic (11.7%) and respiratory (9.4%) conditions. Significant differences concerned hematological comorbidities (CoV2+*ve versus* CoV2-*ve:* 4.6% *versus* 3.2%, respectively; p=0.009).

Overall, 3,711 patients (10.6%) were in the two lower RUBs (non-users and healthy users), while 6,146 (17.6%) were in the high and 3,128 (9%) in the very high RUBs. Compared with CoV2-*ve* cancer patients, those found CoV2+*ve* included a higher proportion of cases in high or very high RUBs (34.4% *versus* 26.3%; p<0.0001).

The patients’ malignancies included 14,861 (42.6%) incident cancers, 5,423 (15.5%) prevalent-active cancers, and 14,645 (41.9%) prevalent-inactive cancers. The proportion of incident cancers was significantly lower among CoV2+*ve* patients (25.6% *versus* 43.1% in CoV2-*ve* cases, p<0.0001).

The most common primary cancer sites involved the breast (18.4%), urinary system (14%), colon-rectum (10.5%), prostate (9.9%), blood (8.9%), and lung (5.5%). This site-specific ranking was much the same for CoV2-*ve* cancer patients, while it differed significantly for CoV2+*ve* patients, who had a higher prevalence of colorectal (12.6%), prostatic (12.3%) and hematological malignancies (11.4%).

Overall, within 12 months before and/or after testing for SARS-CoV-2, 22.3% patients had received chemotherapy and 13.4% had undergone radiotherapy. Both therapies significantly prevailed in the CoV2-*ve* cancer patients (22.6% *versus* 14.0% in the CoV2*+ve* patients, and 13.6% *versus* 6.4%, respectively; p<0.0001).

A median 211 days after testing for the virus (interquartile range 170–248 days), a total of 7,120 patients had died. The proportion of deaths was significantly higher among the CoV2+*ve* patients (n=348; 31.9%) than in the CoV2-*ve* group (n=6,772; 20%; p<0.0001).

### SARS-CoV-2 Infection Risk Factors in Cancer Patients

**Table 2** shows the association of SARS-CoV-2 infection with the explanatory factors selected using the LRM. The risk of infection increased by 1.2% per year of age (OR 1.012; 95%CI 1.007-1.017). While none of the specific comorbidities considered significantly affected the risk of infection, a 1-level increase in RUB was directly associated with SARS-CoV-2 infection (OR 1.14; 95%CI 1.05-1.24).

**Table 2:** Risk of SARS-CoV-2 infection among cancer patients: multivariable logistic regression model.

Variables (number of cancer patients)	Risk of SARS-CoV-2 infection in the population with cancer	
	OR	95%CI
Sex		
Male (17,584)	1.00	–
Female (17,345)	1.09	0.94 – 1.26
Age		
1-year increase	1.012	1.007 – 1.017
Resources Utilization Band		
“1-Band” increase	1.14	1.05 – 1.24
Type of comorbidity		
None (17,609)	1.00	–
Cardiovascular (10,813)	1.06	0.90 - 1.25
Endocrine (3,736)	1.02	0.82 – 1.25
Neurological (2,842)	1.12	0.89 – 1.41
Respiratory (2,256)	1.16	0.89 – 1.50
Gastroenterological (1,927)	0.87	0.64 – 1.17
Hematological (1,121)	1.29	0.92 – 1.76
Renal (1,594)	0.81	0.59 – 1.10
Rheumatologic (1,039)	0.94	0.64 – 1.33
Psychiatric (1,019)	1.00	0.69 – 1.40
Others (2,452)	1.20	0.93 – 1.53
Cancer status		
Prevalent-inactive (14,645)	1.00	–
Incident (14,861)	0.42	0.36 – 0.50
Prevalent-active (5,423)	0.45	0.35 – 0.57
Cancer site		
Colon-rectum (3,658)	1.00	–
Blood (3,101)	1.33	1.03 – 1.72
Prostate (3,452)	1.05	0.82 – 1.36
Breast (6,441)	0.80	0.63 – 1.02
Lung (1,934)	0.82	0.58 – 1.16
Urinary system (4,876)	0.81	0.63 – 1.05
Others (11,467)	0.99	0.81 – 1.23
Treatments		
None (24,664)	1.00	–
Chemotherapy only (5,593)	1.00	0.78 – 1.27
Radiotherapy only (2,471)	0.74	0.50 – 1.05
Chemo and radio (2,201)	0.88	0.60 – 1.26

Patients with incident (OR 0.42; 95%CI 0.36-0.50) or prevalent-active cancer (OR 0.45; 95%CI 0.35-0.57) had a lower risk of infection than patients with prevalent-inactive cancers.

Among cancer sites, the risk of viral infection only rose for hematological malignancies (OR 1.33; 95%CI 1.03-1.72) as compared with colorectal cancer (taken as the reference category because it is the most common cancer among those affecting both sexes).

### Risk of Death in Cancer Patients Positive for SARS-CoV-2

**Table 3** shows the results of the multivariable analysis on mortality risk factors in CoV2+*ve* cancer patients. The risk of death was halved in women (OR 0.50, 95%CI 0.34-0.71) and increased by 7% per year of age (OR 1.07; 95%CI 1.06-1.09).

**Table 3:** Risk of death among CoV2+*ve* cancer patients: multivariable logistic regression model.

Variables (number of cancer patients)	Risk of death in the CoV2+ve population with cancer	
	OR	95%CI
Sex		
Male (543)	1.00	–
Female (547)	0.50	0.34 – 0.71
Age		
1-year increase	1.07	1.06 – 1.09
Resources Utilization Band		
“1-Band” increase	1.13	0.94 – 1.36
Type of comorbidity		
None (456)	1.00	–
Cardiovascular (425)	1.48	0.99 – 2.21
Endocrine (139)	2.09	1.25 – 3.51
Neurological (128)	1.44	0.84 – 2.44
Respiratory (102)	2.87	1.61 – 5.14
Gastroenterological (56)	1.66	0.81 – 3.39
Hematological (50)	3.08	1.49 – 6.50
Renal (54)	3.18	1.58 – 6.49
Rheumatologic (34)	0.86	0.33 – 2.06
Psychiatric (37)	1.93	0.85 – 4.29
Others (99)	1.22	0.66 – 2.21
Cancer status		
Prevalent-inactive (699)	1.00	–
Incident (279)	2.45	1.66 – 3.63
Prevalent-active (112)	2.97	1.63 – 5.42
Cancer site		
Colon-rectum (137)	1.00	–
Blood (124)	1.81	1.01 – 3.25
Prostate (134)	0.82	0.45 – 1.50
Breast (183)	1.32	0.74 – 2.39
Lung (45)	3.55	1.56 – 8.33
Urinary system (127)	0.83	0.46 – 1.48
Others (340)	1.18	0.72 – 1.95
Treatments		
None (903)	1.00	–
Chemotherapy only (117)	1.46	0.82 – 2.59
Radiotherapy only (34)	0.49	0.17 – 1.26
Chemo and radio (36)	4.34	1.85 – 10.50

The risk of a fatal outcome tripled in patients with respiratory, hematological and renal comorbidities and doubled in patients with endocrine comorbidity; the association between mortality and RUB was borderline (OR 1.13; 95%CI 0.94-1.36). Incident and prevalent-active cancers carried a significantly higher risk of death than prevalent-inactive malignancies (OR 2.45; 95%CI 1.66-3.63, and OR 2.97; 95%CI 1.63-5.42, respectively). Compared with colorectal cancer, the risk of death increased significantly for patients with lung cancer (OR 3.55; 95%CI 1.56-8.33) and blood malignancies (OR 1.81; 95%CI 1.01-3.25). It was also four times as high for patients who had been given multimodal chemo-/radio-therapy within 12 months before and/or after testing for SARS-CoV-2 (OR 4.34; 95%CI 1.85-10.50).

## Discussion

As a plausible result of cancer patients’ adherence to lockdown measures, the latest studies did not associate cancer patients with any significantly higher risk of SARS-CoV-2 infection than in the population at large (16, 17).

No population-based studies on cancer patients alone, have compared the clinical-epidemiological profile of CoV2+*ve* as opposed to CoV2-*ve* cancer patients. In the present study on a consecutive cohort of cancer patients tested for SARS-CoV-2 infection, we analyzed the association between the clinical profiles of a consecutive cohort of cancer patients tested for SARS-CoV-2 infection (considering cancer activity status, cancer site, and comorbidities) and their clinical outcomes, such as SARS-CoV-2 infection and death.

No sex-related difference in the infection rate came to light in the cohort of 34,929 cancer patients considered here, despite the well-established prevalence of males contracting the virus in the general population, irrespective of their ethnicity (2, 4, 8).

As expected, the median age of our cancer population was considerably older than that of the general population (with or without cancer) testing positive for SARS-CoV-2 infection. The median age of CoV2+*ve* cancer patients was also 5 years older than that of CoV2-*ve* cancer patients. In keeping with well established information, the multivariable LRM significantly linked increasing age with a higher risk of infection (11, 16, 18, 19).

The LRM model also showed a direct relationship between RUB and risk of infection (3). In the cancer population considered here, the proportion of patients clustering in the high and very-high RUBs (34.4% and 26.3% of CoV2+*ve* and CoV2-*ve* cancer patients, respectively) was far greater than for the regional population as a whole (4.4% in 2019). Adopting RUB as a proxy of comorbidities, our results are in keeping with studies that recognized increasing numbers of comorbidities as a risk factor for SARS-CoV-2 infection (3, 14, 15, 20).

Compared with prevalent-inactive cancers, the risk of infection was more than halved for both incident and prevalent-active cancers. This finding apparently contradicts any greater “susceptibility” to the virus in patients with a (recent) history of malignant disease (such as incident and/or prevalent-active cancers), and differs from the results obtained by Wang and coworkers, who associated an increased risk for viral infection (adjusted OR, 7.14 [95%CI, 6.91-7.39] to patients with a recent cancer diagnosis (12). Our figures, however, as obtained in a homogeneous population living in a region with advanced health care system, could be plausibly interpreted as due to the stricter compliance with social distancing requirements resulting into a lower exposure of recently-diagnosed cancer patients (i.e: incident and/or prevalent-active cancer patients) to the risk of SARS-CoV-2 infection (11, 12, 16, 21).

The prevalence of the different malignancies by site in our study cohort was basically consistent with the proportions documented by the Regional Tumor Registry as at January 1, 2018 (**Table A.3**) (22).

The prevalence of malignancies in the urinary system, blood, prostate and lung may reflect the variability in the epidemiological profiles of the different populations addressed and/or in the case mix at a given local institution. The present results suggest that (non-blood) primary cancer site is not one of the main determinants of the risk of viral infection (11, 12, 18, 19, 21, 23).

Our findings concerning the inverse association between the prevalence of SARS-CoV-2 positivity and recent anticancer therapies are basically in keeping with those regarding the differences between incident or prevalent-active cancer on the one hand and prevalent-inactive cancer on the other: they suggest that recent cancer therapies do not carry a higher risk of viral infection. That said, there is also the possibility that the outbreak of the SARS-CoV-2 pandemic could have led to previously-scheduled anticancer therapies being delayed/canceled (11).

During the time elapsing after testing for the virus (211 days), in keeping with the available information, there were significantly more deaths among the CoV2+*ve* cancer patients (31.9%) than in the CoV2-*ve* group (20%) (4, 12).

When the risk of death was tested with the LRM, male sex and increasing age emerged as significant mortality risk factors in the population of CoV2+*ve* cancer patients.

A recent French study reported a mortality rate of 19% among CoV2+*ve* cancer patients, similar to the 20% observed in the general population (11). These figures, probably resulting from the single-institution French experience, differ considerably from both the higher (32%) mortality rate obtained in the present population-based study and from the (consistent) findings of studies and meta-analyses that associate SARS-CoV-2 infection in cancer patients with consistently higher mortality rates than for COVID-19 patients without cancer (1, 7, 20, 24, 25).

We also found the risk of death significantly associated with both incident and prevalent-active cancers; consistently, a four times risk of death was associated with recent multimodal chemo-radio-therapy (and the related immunodeficiency). As documented by studies comparing cancer *versus* non-cancer patients, lung malignancies were associated with an increased risk of death.

The main strengths of this study lie in: the population-based setting, making the results more representative than those obtained from (mono-)institutional case series and in having considered only cancer patients, thereby potentially excluding (known and unknown) confounding factors deriving from the clinical-biological comparison of individuals with and without cancer.

As for the study’s limitations, the current lack of information on cancer stage curtails (potentially interesting) speculations on the the impact of the cancer status among the determinants of the clinical course of the viral infection (and *vice versa*). Moreover, our distinction between cases of incident and prevalent-active as opposed to prevalent-inactive disease was mainly founded on administrative datasets, so we cannot exclude the occurrence of a (restricted) number of patients being misclassified, possibly leading to the observed differences being underestimated.

## Conclusions

In a large cohort of cancer patients, the risk of SARS-CoV-2 infection was higher for males and increased with age. It was significantly higher for patients with hematological malignancies and those with prevalent-inactive cancer. For CoV2+*ve* cancer patients, having active cancer and having recently received both chemotherapy and radiotherapy significantly raised the risk of death, which increased particularly for lung cancer patients.

## Data Availability Statement

The datasets presented in this study can be found in online repositories. The names of the repository/repositories and accession number(s) can be found below: https://figshare.com/articles/dataset/SARS-COV-2_INFECTION_IN_CANCER_PATIENTS_A_population-based_study/16719997.

## Ethics Statement

This study was approved by Bioethics Committee of the Veneto Regional Authority (protocol number. 245343/2020). Written informed consent for participation was not required for this study in accordance with the national legislation and the institutional requirements.

## Author Contributions

MZ, conceptualization, methodology, writing - original draft, writing - review and editing. SG, data curation, formal analysis, writing - original draft. FA, data curation, methodology. UF, writing - review and editing. AC, conceptualization, writing - review and editing. MR, conceptualization, methodology, writing - original draft, writing - review and editing, supervision. All authors contributed to the article and approved the submitted version.

## Conflict of Interest

The authors declare that the research was conducted in the absence of any commercial or financial relationships that could be construed as a potential conflict of interest.

## Publisher’s Note

All claims expressed in this article are solely those of the authors and do not necessarily represent those of their affiliated organizations, or those of the publisher, the editors and the reviewers. Any product that may be evaluated in this article, or claim that may be made by its manufacturer, is not guaranteed or endorsed by the publisher.

## References

[1] Liang W Guan W Chen R Wang W Li J Xu K. Cancer Patients in SARS-CoV-2 Infection: A Nationwide Analysis in China. Lancet Oncol (2020) 21:335–7. doi: 10.1016/S1470-2045(20)30096-6 PMC7159000 32066541

[2] Dai M Liu D Liu M Zhou F Li G Chen Z. Patients With Cancer Appear More Vulnerable to SARS-CoV-2: A Multicenter Study During the COVID-19 Outbreak. Cancer Discov (2020) 10:783–91. doi: 10.1158/2159-8290.CD-20-0422 PMC7309152 32345594

[3] Yang J Zheng Y Gou X Pu K Chen Z Guo Q. Prevalence of Comorbidities and Its Effects in Patients Infected With SARS-CoV-2: A Systematic Review and Meta-Analysis. Int J Infect Dis (2020) 94:91–5. doi: 10.1016/j.ijid.2020.03.017 PMC7194638 32173574

[4] Kuderer NM Choueiri TK Shah DP Shyr Y Rubinstein SM Rivera DR. Clinical Impact of COVID-19 on Patients With Cancer (CCC19): A Cohort Study. Lancet (2020) 39:1907–18. doi: 10.1016/S0140-6736(20)31187-9 PMC7255743 32473681

[5] Tian Y Qiu X Wang C Zhao J Jiang X Niu W. Cancer Associates With Risk and Severe Events of COVID-19: A Systematic Review and Meta-Analysis. Int J Cancer (2021) 14:363–74. doi: 10.1002/ijc.33213 PMC7404763 32683687

[6] Desai A Sachdeva S Parekh T Desai R. COVID-19 and Cancer: Lessons From a Pooled Meta-Analysis. JCO Glob Oncol (2020) 6:557–9. doi: 10.1200/GO.20.00097 PMC7193801 32250659

[7] Miyashita H Mikami T Chopra N Yamada T Chernyavsky S Rizk D. Do Patients With Cancer Have a Poorer Prognosis of COVID-19? An Experience in New York City. Ann Oncol (2020) 31:1088–9. doi: 10.1016/j.annonc.2020.04.006 PMC7172785 32330541

[8] Wu Z McGoogan JM. Characteristics of and Important Lessons From the Coronavirus Disease 2019 (COVID-19) Outbreak in China: Summary of a Report of 72 314 Cases From the Chinese Center for Disease Control and Prevention. JAMA (2020) 32:1239–42. doi: 10.1001/jama.2020.2648 32091533

[9] Guan WJ Ni ZY Hu Y Liang WH Ou CQ He JX. Clinical Characteristics of Coronavirus Disease 2019 in China. N Engl J Med (2020) 382:1708–20. doi: 10.1056/NEJMoa2002032 PMC7092819 32109013

[10] Lee LYW Cazier JB Starkey T Briggs SEW Arnold R Bisht V. COVID-19 Prevalence and Mortality in Patients With Cancer and the Effect of Primary Tumour Subtype and Patient Demographics: A Prospective Cohort Study. Lancet Oncol (2020) 21:1309–16. doi: 10.1016/S1470-2045(20)30442-3 PMC7444972 32853557

[11] Basse C Diakite S Servois V Frelaut M Noret A Bellesoeur A. Characteristics and Outcome of SARS-CoV-2 Infection in Cancer Patients. JNCI Cancer Spectr (2021) 5:pkaa090. doi: 10.1093/jncics/pkaa090 33604509 PMC7665636

[12] Wang Q Berger NA Xu R. Analyses of Risk, Racial Disparity, and Outcomes Among US Patients With Cancer and COVID-19 Infection. JAMA Oncol (2021) 7:220–7. doi: 10.1001/jamaoncol.2020.6178 PMC7729584 33300956

[13] The Johns Hopkins University Bloomberg School of Public Health, Health Services Research Development Center. The Johns Hopkins ACG System: Installation and Usage Guide, Version 9.0. Baltimore (MD): Johns Hopkins University Bloomberg School of Public Health (2009).

[14] Dotto M Avossa F Schievano E Basso C Netti ST Fedeli U. Rapporto Epidemiologico Sulle Malattie Croniche in Veneto. Dati Anno (2019). Available at: https://www.ser-veneto.it/public/Rapporto_epidemiologico_malattie_croniche_Veneto_2019.pdf.

[15] Lee WC. Quantifying Morbidities by Adjusted Clinical Group System for a Taiwan Population: A Nationwide Analysis. BMC Health Serv Res (2008) 8:153. doi: 10.1186/1472-6963-8-153 18644140 PMC2492856

[16] Rugge M Zorzi M Guzzinati S. SARS-CoV-2 Infection in the Italian Veneto Region: Adverse Outcomes in Patients With Cancer. Nat Cancer (2020) 784–8. doi: 10.1038/s43018-020-0104-9 35122051

[17] Mangone L Gioia F Mancuso P Bisceglia I Ottone M Vicentini M. Cumulative COVID-19 Incidence, Mortality, and Prognosis in Cancer Survivors: A Population-Based Study in Reggio Emilia, Northern Italy. Int J Cancer (2021) 149(4):820–6. doi: 10.1002/ijc.33601 PMC8250826 33861870

[18] Yang K Sheng Y Huang C Jin Y Xiong N Jiang K. Clinical Characteristics, Outcomes, and Risk Factors for Mortality in Patients With Cancer and COVID-19 in Hubei, China: A Multicentre, Retrospective, Cohort Study. Lancet Oncol (2021) 21:904–13. doi: 10.1016/S1470-2045(20)30310-7 PMC7259917 32479787

[19] Tian J Yuan X Xiao J Zhong Q Yang C Liu B. Clinical Characteristics and Risk Factors Associated With COVID-19 Disease Severity in Patients With Cancer in Wuhan, China: A Multicentre, Retrospective, Cohort Study. Lancet Oncol (2020) 2:893–903. doi: 10.1016/S1470-2045(20)30309-0 PMC7259911 32479790

[20] Wang D Hu B Hu C Zhu F Liu X Zhang J. Clinical Characteristics of 138 Hospitalized Patients With 2019 Novel Coronavirus-Infected Pneumonia in Wuhan, China. JAMA (2020) 323:1061–9. doi: 10.1001/jama.2020.1585 PMC7042881 32031570

[21] Lièvre A Turpin A Ray-Coquard I Le Malicot K Thariat J Ahle G. Risk Factors for Coronavirus Disease 2019 (COVID-19) Severity and Mortality Among Solid Cancer Patients and Impact of the Disease on Anticancer Treatment: A French Nationwide Cohort Study (GCO-002 CACOVID-19). Eur J Cancer (2020) 141:62–81. doi: 10.1016/j.ejca.2020.09.035 33129039 PMC7543792

[22] The Tumours in Veneto. Available at: https://gecoopendata.registrotumoriveneto.it/prevalenza.php.

[23] Wang B Huang Y. Which Type of Cancer Patients are More Susceptible to the SARS-COX-2: Evidence From a Meta-Analysis and Bioinformatics Analysis. Crit Rev Oncol Hematol (2020) 153:103032. doi: 10.1016/j.critrevonc.2020.103032 32599375 PMC7295508

[24] Zhang L Zhu F Xie L Wang C Wang J Chen R. Clinical Characteristics of COVID-19-Infected Cancer Patients: A Retrospective Case Study in Three Hospitals Within Wuhan, China. Ann Oncol (2020) 31:894–901. doi: 10.1016/j.annonc.2020.03.296 32224151 PMC7270947

[25] Zhang H Han H He T Labbe KE Hernandez AV Chen H. Clinical Characteristics and Outcomes of COVID-19-Infected Cancer Patients: A Systematic Review and Meta-Analysis. J Natl Cancer Inst (2021) 113:371–80. doi: 10.1093/jnci/djaa168 PMC7665647 33136163

