# Supplementary material for: SARS-CoV-2 Infection in Cancer Patients: A Population-Based Study
Source: Front Oncol. 2021 Oct 11;11:730131. doi: 10.3389/fonc.2021.730131 (PMC8542867; doi:10.3389/fonc.2021.730131)
Supplement: Supplementary file 1 [file Table_1.docx]

**APPENDICES**

| **Table A.1**  Selected Expanded Diagnosis Clusters (EDCs) | |
| --- | --- |
| **ALL** | **Allergy** |
| ALL05 | Asthma, with status asthmaticus |
| **CAR** | **Cardiovascular** |
| CAR03 | Ischemic heart disease (excluding acute myocardial infarction) |
| CAR04 | Congenital heart disease |
| CAR05 | Congestive heart failure |
| CAR06 | Cardiac valve disorders |
| CAR07 | Cardiomyopathy |
| CAR09 | Cardiac arrhythmia |
| CAR10 | Generalized atherosclerosis |
| CAR11 | Disorders of lipoid metabolism |
| CAR12 | Acute myocardial infarction |
| CAR13 | Cardiac arrest, shock |
| CAR15 | Hypertension, with major complications |
| **END** | **Endocrine** |
| END04 | Hypothyroidism |
| END07 | Type 2 diabetes, w/ complication |
| END09 | Type 1 diabetes, w/ complication |
| **EYE** | **Eye** |
| EYE01 | Ophthalmic signs and symptoms |
| EYE07 | Conjunctivitis, keratitis |
| EYE13 | Diabetic retinopathy |
| **GAS** | **Gastrointestinal/Hepatic** |
| GAS02 | Inflammatory bowel disease |
| GAS04 | Acute hepatitis |
| GAS05 | Chronic liver disease |
| GAS06 | Peptic ulcer disease |
| GAS11 | Acute pancreatitis |
| GAS12 | Chronic pancreatitis |
| GAS13 | Lactose intolerance |
| **GTC** | **Genetic** |
| GTC01 | Chromosomal anomalies |
| GTC02 | Inherited metabolic disorders |
| **HEM** | **Hematologic** |
| HEM01 | Other hemolytic anemia |
| HEM05 | Aplastic anemia |
| HEM06 | Deep vein thrombosis |
| HEM07 | Hemophilia, coagulation disorder |
| HEM08 | Hematologic disorders, other |
| HEM09 | Sickle cell disease |
| **INF** | **Infections** |
| INF01 | Tuberculosis infection |
| INF04 | HIV, AIDS |
| INF08 | Septicemia |
| **NUR** | **Neurologic** |
| NUR05 | Cerebrovascular disease |
| NUR06 | Parkinson's disease |
| NUR08 | Multiple sclerosis |
| NUR09 | Muscular dystrophy |
| NUR11 | Dementia and delirium |
| NUR20 | Central nervous system infections |
| **NUT** | **Nutrition** |
| NUT02 | Nutritional deficiencies |
| NUT03 | Obesity |
| **PSY** | **Psychosocial/Mental Health** |
| PSY01 | Anxiety, neuroses |
| PSY02 | Substance use |
| PSY03 | Tobacco use |
| PSY09 | Depression |
| PSY12 | Bipolar disorder |
| **REN** | **Renal** |
| REN01 | Chronic renal failure |
| REN03 | Acute renal failure |
| **RES** | **Respiratory** |
| RES02 | Acute lower respiratory tract infection |
| RES03 | Cystic fibrosis |
| RES04 | Emphysema, chronic bronchitis, COPD |
| RES08 | Pulmonary embolism |
| RES10 | Respiratory failure |
| **RHU** | **Rheumatologic** |
| RHU01 | Autoimmune and connective tissue diseases |
| RHU02 | Gout |
| RHU03 | Arthropathy |
| RHU04 | Raynaud's syndrome |
| RHU05 | Rheumatoid arthritis |
| **SKN** | **Skin** |
| SKN12 | Psoriasis |
| **TOX** | **Toxic Effects and Adverse Events** |
| TOX01 | Toxic effects of nonmedicinal agents |
| TOX02 | Adverse effects of medicinal agents |
| TOX03 | Adverse events from medical/surgical procedures |

**Table A.2**

Resource Utilization Bands (RUBs) for the whole resident population of the Veneto region (north-east Italy) where the study was conducted (year 2019).

|  | Resource Utilization Bands (RUBs) | **%** |
| --- | --- | --- |
| 0 | Non-users | 22·3 |
| 1 | Healthy users | 33·9 |
| 2 | Low | 19·4 |
| 3 | Moderate | 19·9 |
| 4 | High | 3·3 |
| 5 | Very high | 1·1 |

**Table A.3** Prevalence of invasive cancers by site in the resident population of the Veneto region considered in the present study (years: January 2007 - December 2017)

| **Primary site of malignancy** | **N** | **%** |
| --- | --- | --- |
| Breast | 40115 | 24·1 |
| Prostate | 28204 | 16·9 |
| Colon-rectum | 21949 | 13·2 |
| Urinary system | 21549 | 12·9 |
| Blood | 13671 | 8·2 |
| Lung | 5726 | 3·4 |
| **Total** | 166435 | 100 |
